# Anæsthetizing Sensitive Dentine

**Published:** 1894-08

**Authors:** W. H. Van Deman


### Anæsthetizing Sensitive Dentine.

BY W. H. VAN DEMAN, D.D.S.

Read before the 38th, Annual Meeting of the Michigan Dental Association.

Much has been said by the dental profession upon the means of obtunding the sensibility of the dentine. It is a matter which is of the greatest importance for the fear of pain during the excavation of cavities is, probably, the most common cause for neglect of the teeth.

If we are to eliminate pain from an operation, we should first study its cause and the means by which it is transmitted to the seat of realization.

When we examine the dentine under the microscope, we see that from the pulp cavity to the periphery, the tissue is perforated by small tubes or canals having distinct walls ; each tube starting by an open circular mouth upon the surface of the pulp cavity, radiating in an undulating course, giving off many branches which freely anastomose or communicate with each other. They terminate either by gradually fading out, by anastomosis, by ending in loops, or in the interglobular spaces. It has been thought by some that these tubes pass into the enamel and cementum, but this has not yet been proved.

Each tube has a definite wall or lining that may be demonstrated, even in fossil teeth. These tubes are not mere bony canals in the matrix, but each tube is lined with a definite, delicate, and yet indestructible structure, the dentinal sheath of Neumann. Rose has proven this sheath to be nothing more than the remains of uncalcified dentinal ground-substance which has experienced some chemical change, by which it has become resistant to acids and alkalies.

What are the contents of these tubules ? It is now definitely known that on the inner surface of the dentine, or better, on the outer surface of the pulp, we have a layer of highly differentiated connective tissue cells, called the odontoblasts, and that through the activity of these cells the dentine is deposited. From these odontoblasts, we find protoplasmic processes of various lengths, extending up into the dental tubules, and here constitute the so-called Tomes dentinal fibers. Some have thought these fibers to come from cells deeper seated in the pulp tissue, below the odontoblasts, but Rose has shown, and his work has been accepted by V. V. Ebner and others, that Tomes fibers are simply protoplasmic processes of the highest differentiated connective tissue cells, the odontoblasts.

Can such processes transmit sensation? It has been thought they can and do. By some the odontoblast has been likened to the amoeba. The amoeba is protoplasm which resembles undifferentiated protoplasm of higher forms; its protoplasm has the power of assimilation, conductivity and reproduction. The odontoblast has no such power, they can not properly be compared at all. One of the most interesting and important facts is, that there is only a single layer of odontoblasts which remain active throughout the life of the tooth.

We have certainly no reason to believe that the protoplasmic processes of the odontoblasts can conduct sensations. Nowhere in the body does a connective tissue cell have such a function, yet there is no doubt but what the sensitiveness of the dentine is due to the contents of the tubules.

May we not have something more than a protoplasmic process enclosed within each of these tubules? Boll, who has probably done the best work on the nerve ending of the tooth, has traced the fibrillæ from the pulp up between the odontoblasts, and believes they may extend on into the tubuli. To me, this is the rational view. I see no reason why we should not expect such an ending of the nerves in the tooth. There is much work yet to be done on the peripheral distribution of the nervous system, however, and it would be unwise to assume that we know all about the nerve structure of the tooth.

Sensitive Dentine is generally physiological, but we know that it is in some instances purely pathological, and in such cases we have what may be called an inflamed or hypersensitive condition.

The term inflammation has been objected to by some, because all the phenomena of inflammation, as it appears in other tissues, are not present, or at least, are not perceptible. Redness, heat, pain and swelling are the four signs distinctive of inflammation, but it is not essential that these should all exist to have inflammation. In inflamed or hypersensitive dentine we always have pain, and may have redness and heat, although the latter may not be perceptible, but owing to the dense structure of the dentine, never swelling.

Some have said "We can not have inflammation of the dentine, because the dentine has no circulation." But the dentine is a living tissue, and all living tissue requires constant nourishment, and is constantly giving off waste products.

How is the dentine nourished and what becomes of its waste products? As yet I have been unable to determine. According to Townsend and others, "We know that the largest of the tubuli are far too small to admit the blood corpuscles, but there is no reason why the blood plasma, or some fluid having like properties, should not enter the tubuli, supply the structure with the necessary nourishment, gather up the waste products, and pass out, by which a circulation is established in each tubule directly connected with that of the pulp."

If this be true, it is easy to explain why it is that we find the dentine of a tooth in which the pulp has, from any cause, become inflamed so fearfully sensitive. If the pulp be treated and restored to its normal condition the sensibility of the dentine is reduced. As to whether either of the terms, inflammation or hypersensitiveness of the dentine, are scientifically correct I have my doubts; of the two, I prefer the latter.

The methods of obtunding sensitive dentine may be classed under three heads: Physical Processes, Medications and Instrumentation.

As Physical Processes we may consider the application of heat and cold. We all know that, oftentimes, by the simple drying out of the dentine with warm air its sensibility is greatly reduced. If we use absolute alcohol, then warm air, this is accomplished much more readily and effectually. When we apply heat we do nothing more than dehydrate the dentine, and just to the extent that we dry up the contents of the tubuli will the normal function of the fiber be reduced. Some have suggested drying out the tubuli in this manner, then filling them with some one of the essential oils, believing that in this way they could prolong the period of insensibility. I believe this wholly unnecessary and dangerous, to the extent that the tooth may be discolored by the deposit from the coloring matter of the oil used.

The cold-producing agents, such as ether spray, chloride of ethyl and chloride of methyl, act upon the dentine by freezing up the contents of the tubuli, thus obtunding their sensibility. The chloride of methyl is probably the best one of these agents. In the cavity to be treated should be placed a piece of cotton saturated in glycerine, then the spray thrown on the cotton. This will lessen the pain considerably, although all cold-producing agents, while effective, are more or less painful.

As Medications we may use caustics (potential caustics) as nitrate of silver, chloride of zinc, carbolic acid and its combinations, as Robinson's remedy and Black's 1, 2, 3 solution.

Nitrate of silver, when applied to sensitive dentine, acts on the contents of the tubuli, destroying its vitality to the extent of the combination which takes place. The objections to its use, which I believe sufficient to exclude it altogether, are the discoloration it causes and its superficial action, being escharotic rather than caustic.

Chloride of zinc, while painful, is a most valuable obtunding agent. Its painful action may be modified by bathing the sensitive surface, prior to the application of the chloride, with chloroform, tincture of aconite or atropine solution. It is very penetrating, and in no case should be used where there is danger of irritating the pulp of the tooth.

Carbolic acid being an antiseptic as well as an escharotic, makes a very valuable agent as an obtundent. Often exceedingly sensitive dentine can be relieved by simply applying carbolic acid, and drying out with hot air. The action is much more rapid and effective, however, if the drug be combined with either alcohol, chloroform or ether. Robinson's remedy, composed of equal parts of carbolic acid and caustic potash, and Black's 1, 2, 3 are often very effective. All of these caustics act by simply coagulating the contents of the tubuli.

Instrumentation, or the use of thoroughly-sharpened instruments, I believe to be the most important of all the methods of obtunding sensitive dentine. Sharp excavators skillfully used will, in many cases, be all that is needed, even retaining forms may be made with the excavators in extreme cases. When the bur in the engine is used it should be sharp and the engine run slowly so as to prevent the generation of excessive heat, which will always cause great pain. Under this head also might be considered the matter of gaining the perfect confidence of the patient. This is, no doubt, of great importance.

### DISCUSSION.

C. B. Blackmar, D.D.S.: About a month ago Dr. Hoff called at my office and informed me that the entire subject to be presented at our meeting here this year would be "Painless Dentistry."

He also said that one of the papers to be read was on the subject of "Obtundents for Sensitive Dentine" from purely a theoretical standpoint, and he desired a practical, operating dentist to open the discussion and asked if I would do it? As I am simply to open the discussion I am going to do so from a practical, business standpoint.

In regard to the paper just read by Dr. Van Deman I would like to say that if any dentist with good common sense will sit down and have the contents of the tubuli frozen with the agents mentioned I am sure he will not call it painless dentistry. And I am quite sure that the dentist who uses, as the paper states, Robinson's remedy, chloride of zinc, oil cassia, etc., will wish for something better before he can call the excavating of dentine painless dentistry. To my mind, practically, the excavating of diseased dentine with an obtundent is a very different thing from excavating healthy dentine, and, by the way, a patient with a bad bringing up combined with a streak of ugliness and a bad temper will, usually derive very little or no benefit from local obtundents. There is one thing I wished to say last night, I will say it now, and that is, in regard to a physician giving chloroform, etc., for us. I never allow one to do it in *my* office but in *his*, and if anything goes wrong, or unpleasant happens, or the patient dies, the victim is in the physician's office and *not* mine. The physician has the whole responsibility.

Last summer I saw a patient undergoing an operation by a dentist using a so-called "Painless Process." She said it did not hurt. Her friend with her, a patient of one of the best dentists in Detroit, said to her as she sat in the chair, "Then I am going to ask Dr. —— if he uses anything now he never did for me before?" This Detroit dentist's answer to her a month later was that it was a fraud, a fake, a humbug, that there was nothing one could put into or around a tooth that would make it hurt less when operated upon. Now, if this statement is so, I am surprised that this subject should be even mentioned by Dr. Hoff at our State Meeting, for we are not supposed to discuss fakes.

Dr. Case once told me that it was a fact that so-called quacks were the first to "catch on to" new remedies and schemes before the regular practitioners did, and these remedies would generally be called fakes at first. A patient told me this summer that she had cocaine applied to her gums in New York City in 1885 to relieve pain from ligatures, etc.

When was it first taught in our dental colleges? Why is it that young dental graduates do not know about some of these things, or all of them, before the quacks, so-called? I think they should be ready to meet them instead of being annoyed, and made to cope with them.

It has been said that people who are ill often get well by the aid of medicine. Some get well without any medicine, and others get well in spite of medicine, and so it is in regard to diseased teeth, that some are filled painlessly by the aid of medicine, some without any medicine and others in spite of medicine, and some are cleaned out and filled with a good deal of pain in spite of all we can do, at present, to avoid it. And some patients are so sensitive in regard to their teeth that scraping on the rubber dam clamp causes them pain. Obtundents for sensitive denine are of little use in such cases. However, it is a fact that certain drugs rightly used will obtund sensitive dentine, so much so, at least, that the extracting can be done with less pain than without, and patients will often say that it was entirely painless, even when before operating the cavity could scarcely be touched without pain.

The right use of any drug is a nice thing. So also it is a satisfactory procedure to use one's patients right. This makes me think of the answer a professor in the State Normal School gave a boy who asked him if there were not two ways to spell a certain word. His answer was. "Yes, a right way and a wrong way."

Did any of you ever notice how some dentists adjust the velvet rubber dam? Is it painless? Did any of you ever have that soft floss silk slipped in between your teeth down on to and into your gum by one of those dentists who does not believe in so-called "Painless Dentistry?" Was it painless? Could it have been made less painful? I think it could. And the rubber dam clamp—did you ever feel one? And yet Dr. Rhein, of New York, in the *Dental Cosmos*, says he is sorry for the dentist who cannot adjust one painlessly. Did any of you ever let some brother dentist try to find an exposed pulp with an instrument just big enough to fit into the exposure and make pressure on the nerve? Did you call that painless? Did you ever see a dentist trying to obtund sensitive dentine by drawing in alcohol vapor from a lamp and blowing it out into the cavity with a chip blower, producing hot and cold blasts alternately, after medicating the cavity with oil cassia, etc., and the dentist wondering why the drug did not work? I have seen a dentist take an excavator to put arsenic into one tooth, the pulp of which he wanted to destroy, and use the same excavator, uncleaned, to clean out another cavity and wonder why that tooth pained afterward. Usually in such cases, if they are reported, the name, age, nationality, sex, color of hair, etc., would be given, but the arsenic would not be mentioned.

"Delicacy of manipulation is what must be acquired by a dentist to be successful," says Dr. Geo- H. Cushing, of Chicago. It includes nearly everything a dentist has to do. Imaginė a dentist trying an obtundent—a drug—on sensitive dentine, and then cutting the dentine with a dull bur. Do you think he will regard the obtundent successful? Or the patient think differently from him? A friend of mine, a fine operator, a professor in a dental college, often would adjust a rubber dam, put in an obtundent in the cavity of a tooth and leave the patient while he shaved himself in his laboratory, wrote a letter or read a chapter or so, and upon his return wonder why the patient called the whole operation painful. I once saw a professor in a dental college tell a lady to hold perfectly still as he was not going to hurt her at all and at the same time he took a chisel-shaped excavator and exposed two pulps. After her scream he said: "Why, did that hurt you?" "Mercy, yes," she said. "That's good, I wanted it to. I had to do that way to treat them," the professor replied. Now, do you think that woman could be made to believe and feel that that man would do work for her, or even look at a tooth without pain to her?

Did any of you ever have an aching sore tooth and be obliged to have your assistant or student, in whom you had little faith, dig or probe into it to see if he could tell, for you, what made it ache? Did it not seem to you that every move he made went direct into the pulp, when in reality he might have been scraping on the rubber-dam clamp simply? But how differently you sat in the chair a few days after when you had gone to a neighbor dentist, in another city perhaps, one in whom you had perfect confidence that he would do just what was right and be accurate and careful. You all know of the many cases on record of death from fright and you all know the influence of one's mind over the body during any trying ordeal. A clipping will, perhaps, make my meaning clearer.

### IMAGINARY PAIN—A CURIOUS EXPERIMENT AT THE PERFORMANCE OF A SURGICAL OPERATION.

"The pain experienced in surgical operations is largely in the imagination of the patient," remarked Dr. W. C. Tousley, of Kansas City. "I once had occasion to perform an operation on a man where it became necessary for me to use the knife twice. After the first part of the operation was over the man declared he could not stand any more, as the pain was more than he could bear. Convinced that the suffering was more imaginary than real, I resorted to this ruse: I called in my assistant and told him to tie a bandage over the man's eyes, which I said was saturated with a soothing lotion which would allay the pain. I then told my assistant to grasp the man's hands and hold them firmly. Then I told the patient to lie perfectly still, and when I said 'Ready!' to prepare himself for the ordeal. I then went to work and in a short time finished the operation, the man remaining perfectly quiet all the time. After the operation was completed I called out 'Ready!' at the same time barely touching the wound with the handle of the knife. The man let out such a roar as I never heard before, and declared I was killing him, struggling with might and main to get free. When told that the operation had been finished without his realizing it, he would scarcely believe it."

Mind-curists or Christian Scientists believe pain can be entirely relieved by putting the mind in a certain condition.

It has been said that a dentist should have a tooth filled himself once a week in order to treat his patients decently—and I think so, too. Dentists generally are not as careful as they ought to be. They forget many times that they are working on living tissue. I think "Painless Dentistry" should be called dentistry with less pain than usual. So-called "Painless Dentistry" I think has done a good deal toward making dentists in general think more about being careful. A patient noticing that a dentist is trying to relieve any little pain and doing little things accurately will have confidence in that dentist that when it does hurt a good deal he is doing his best to have it hurt as little as possible. Why, I believe a dentist should try to have his patients have as much confidence in him in this respect as the S. S. White Co. tries to have us dentists have in the superiority of their burs. But whether a dentist should advertise his work to his patients in the way the S. S. White Co. does its goods to us, I suppose would be a question to some dentists.

This subject of "Painless Dentistry" has placed upon the market a large number of obtundents—our dental depots and agents advising us to use this and that.

Dr. Hoff says he is continually getting letters from students who have graduated and gone to practicing and who have encountered quacks here and there who were extracting teeth painlessly and asking no fees, and it hurt the recent graduates' practice so much that they had nothing to do. Now, who is to blame? If a graduate cannot convince the people that he can pull teeth better than a quack, then he is to blame. If a quack can pull teeth better, and has a right to pull teeth according to the laws of this State, he is not to blame and the people are not to blame for going to him. But if the dental profession, or we will say this body of dentists, know that the quack does mischief to the people rather than good there is a way of letting the people know it. There is a way of getting laws so that the young graduate will be rightly appreciated. But it is not the way to make the young graduate lie down and keep quiet until such a time comes. For at present he is obliged, in a business way, to try to get a practice under laws which allow quackish methods. There was a time when dentists in this State were annoyed by unprepared men practicing dentistry. It is not so now. Dentists used to be constantly called upon to give patients "vitalized air," but I have scarcely heard the words used since the time Professor Watling's resolution was before this association and was copied into the daily presses and so educated the people, showing them that "vitalized air" was simply chloroform mixed with nitrous oxide gas; showing the people, the patients, that they were being fooled by the quacks, so-called, and the people appreciated it. And so I say about these young men who are so annoyed, that I think they should be allowed to show or be helped to show the people, their patients, that the quack is wrong. If the quack is wrong (and he should be rather than the regular practitioner) and it can be shown clearly and positively to the people, it will not be long before the young graduate will have the practice he deserves. Business principles mixed with professional principles would remedy it all.

Dr. L. E. Custer: One point I would like to speak of—the part imagination plays in dental operations, especially the cutting of sensitive dentine. Imagination is a subjective faculty. We have to deal with a mental activity. The impressions are brought to the mind of the patient through the several senses and the pain felt through these agencies is, for the time being, most real; the sense of sight, for instance. A grand display of instruments in dentist's office is all very well in its way. Such instruments enable him to do excellent work and are essential, but they should be kept out of sight of the patient. The sense of smell has its influence, too. The odor of iodine, for instance, will bring to the mind of the patient painful memories; the odor of carbolic acid and creosote have a like effect upon the minds of some patients. The sense of hearing is another agency through which imaginary pain is felt. I think that in the case of ladies, particularly, who are accustomed to the quiet of the household, the pain is aggravated by the noisy surroundings in a dentist's office.

Personal magnetism should be exercised as much as possible by the operator. He should treat his patients as though they were living, sensitive human beings. He should inspire them with confidence in himself and his ability to perform the required operation. He should keep them as quiet as possible and guard against all irritation to the senses of sight, hearing and smell.

Dr. Barbour: I think the best results are obtained by running the engine slowly. Use a smooth-running engine, and a sharp bur, bearing on lightly. One has better control of the bur, and certainly there is less friction and less pain than when more pressure is used.

Dr. Lathrop: I have always thought it was best to run the engine rapidly, using a sharp bur and light pressure.

Dr. Field: I heartily endorse what the last gentleman has said. I use a very sharp bur—a dull one I throw away—and run the engine just as fast as I can, just touching the tooth, and lifting the bur from time to time so that if any undue heat be generated, it has time to cool in lowering.

Dr. Moore: I have never been able to satisfy myself that a rapid-running engine was better than one run slowly. I think a fairly good size bur, well sharpened and run rather slowly, with a good pressure will do better work. Rapid running causes more friction and more heat, and it is the heat I think that causes the pain.

DR. WATLING: It is, I think, a very hard matter to decide which is best to use, a rapid or a slow movement of the engine. I have had a great deal of experience in excavating teeth, perhaps more than anybody else here, and I have never fully decided as to which is better. But I am rather inclined to agree with Dr. Moore. An excavator well sharpened does the work with less pain than the bur. Why? Because there is no friction, and personally I think I prefer it to the bur.

DR. PARKER: I believe that pain is always the result of pressure upon some nerve. Whichever method produces the least pressure will cause the least pain. Another element is time. The excavator produces less pressure, and hence less pain, because less time is taken.

DR. SIDDALL: Much of the pain felt during dental operations is mental rather than physical. If we have a patient who thinks he is going to be hurt, that person invariably will be hurt. If the operation can only be done so quietly and quickly that he does not realize what is being done, there is not apt to be so much pain. The engine makes more noise than the excavator, and that I believe is why more pain is felt when it is used. I am confident that nine-tenths of the pain experienced is imaginary. Of course a great many things do hurt, and hurt badly, but in the majority of cases the pain is mental rather than physical.

